# Wavefront sensing at a high-repetition-rate X-ray free-electron laser

**DOI:** 10.1107/S1600577526005898

**Published:** 2026-07-15

**Authors:** Trey Guest, Patrik Vagovič, Luigi Adriano, Johan Bielecki, Sarlota Birnsteinova, Rita Graceffa, Jayanath Koliyadu, Kaye Morgan, Hiiro Moriyama, Daniel Mosko, Grant van Riessen, Kristian Sabol, Tokushi Sato, Peter Szeles, Zane Taylor, Jozef Uličný, Richard Bean, Adrian P. Mancuso, Brian Abbey

**Affiliations:** a European XFEL Holzkoppel 4 22869Schenefeld Germany; b La Trobe Institute for Molecular Science La Trobe University Bundoora VIC3086 Australia; c Department of Mathematical and Physical Sciences, School of Engineering, Computing and Mathematical Sciences La Trobe University Bundoora VIC3086 Australia; d Center for Free-Electron Laser Science (CFEL) DESY Hamburg Germany; e School of Physics and Astronomy Monash University Clayton VIC3800 Australia; f Department of Biophysics, University of P. J. Šafárik, Košice, Slovakia; g Department of Materials Science and Engineering Stanford University Stanford CA94305 USA; h Diamond Light Source Harwell Science and Innovation Campus DidcotOX11 0DE United Kingdom; DESY, Germany

**Keywords:** wavefront sensing, X-ray free-electron lasers, X-ray speckle tracking

## Abstract

Wavefront characterization is essential for diagnosing, interpreting and mitigating performance limitations at X-ray free-electron lasers (XFELs). However, the dramatic increase in thermal load and data throughput at high repetition rates makes established wavefront characterization methods difficult to implement effectively. Here, we demonstrate that X-ray speckle arising from beamline optics can enable fast, robust and sensor-free wavefront metrology at next-generation XFEL facilities. Combining this approach with a statistical formulation of X-ray speckle tracking, we quantify local wavefront fluctuations within pulse trains at the European XFEL. We find that shot-to-shot wavefront fluctuations are predominantly planar phase tilts with distinct statistical signatures across intra- and inter-train timescales. The dominant source of wavefront error is periodic at frequencies consistent with known mechanical oscillation modes of the photon transport optics, while intra-train fluctuations correlate with instabilities in the electron bunch trajectory. Our results establish a practical framework for high-repetition-rate wavefront characterization and diagnostics at next-generation XFELs.

## Abstract 2

We demonstrate a fast, robust and sensor-free approach to X-ray speckle tracking that enables shot-to-shot wavefront characterization at high-repetition-rate X-ray free-electron lasers. Our measurements reveal a hierarchy of wavefront fluctuations indicative of instabilities in the accelerator and beamline optics at the European XFEL.

## 1. Introduction

X-ray free-electron lasers (XFELs) combine high pulse intensity, transverse coherence and ultrashort pulse durations to enable the investigation of matter on atomic length scales (Chapman *et al.*, 2006 ▸) and attosecond timescales (Yan *et al.*, 2024 ▸). The emergence of superconducting accelerators with vastly improved duty cycles has marked the advent of the *next generation* of XFELs, which has been characterized by the commissioning of several facilities capable of operating at high repetition rates (Altarelli, 2006 ▸; Decking *et al.*, 2020 ▸; Raubenheimer, 2018 ▸; Zhao & Ding, 2024 ▸). As the first of these next-generation facilities to operate in the hard X-ray regime, the European XFEL is driving fundamental research in structural biology (Grünbein *et al.*, 2018 ▸; Gisriel *et al.*, 2019 ▸; Sobolev *et al.*, 2024 ▸), materials science (Vagovič *et al.*, 2019 ▸; Shayduk *et al.*, 2022 ▸; Jo *et al.*, 2025 ▸) and high energy density physics (Laso Garcia *et al.*, 2024 ▸; Mercadier *et al.*, 2024 ▸; Kraus *et al.*, 2025 ▸).

The radiation produced at the European XFEL is inherently stochastic (Geloni *et al.*, 2010 ▸). Shot-to-shot fluctuations of the X-ray pulse wavefield are a natural consequence of the self-amplified spontaneous emission (SASE) process (Saldin *et al.*, 2000 ▸), and these fluctuations are compounded by operational instabilities such as mechanical vibrations (Sinn *et al.*, 2022 ▸), thermal drifts (Zhang *et al.*, 2025 ▸) and electronic noise (Floettmann, 2015 ▸). The transition to high repetition rates coincides with a dramatic increase in the thermal and operational load on the accelerator and beamline infrastructures, which has led to emergent instabilities of the X-ray beam on newly accessible timescales (Aquila *et al.*, 2015*a* ▸; Hellert *et al.*, 2017*a* ▸; Hellert & Schmidt, 2018 ▸; Yang *et al.*, 2022 ▸). The combined effect of these intrinsic and extrinsic factors culminates in pulse statistics which fluctuate across timescales spanning attoseconds to days (Czwalinna *et al.*, 2021 ▸).

As a direct consequence of the short duration and high spatial coherence of XFEL pulses, snapshots of instabilities in the accelerator and beamline optics are observed as shot-to-shot fluctuations in the X-ray pulse wavefront (Rutishauser *et al.*, 2012 ▸). Diagnosing, interpreting and mitigating these fluctuations is critical to maximizing experimental outcomes, particularly at next-generation facilities, where many high-repetition-rate experiments are acutely sensitive to the coherence of the X-ray beam (Aquila *et al.*, 2015*b* ▸; Nakano *et al.*, 2018 ▸; Sun *et al.*, 2018 ▸; Cao *et al.*, 2020 ▸; Dallari *et al.*, 2021 ▸; Buakor *et al.*, 2022 ▸). Fluctuations of the XFEL source and optics can limit the reproducibility of imaging, diffraction and spectroscopy experiments more generally, due to subsequent fluctuations in pulse intensity and spectrum which can arise during photon transport (Neutze, 2014 ▸; Coe & Fromme, 2016 ▸; Gorel *et al.*, 2021 ▸; Biednov *et al.*, 2023 ▸). If left uncorrected, these fluctuations degrade experimental outcomes by increasing shot-to-shot variance in recorded data, reducing signal-to-noise ratios, and introducing artefacts during data merging and normalization.

Developing experimental and analytical strategies to mitigate these fluctuations requires wavefront diagnostics that can be applied routinely at high repetition rates. While Hartmann sensing (Keitel *et al.*, 2024 ▸), grating interferometry (Liu *et al.*, 2018 ▸; Seaberg *et al.*, 2019 ▸) and ptychography (Schropp *et al.*, 2013 ▸) provide established routes to wavefront characterization, their application at high repetition rates is technically demanding and often impractical due to stringent sensor requirements (Samoylova *et al.*, 2009 ▸), restrictive experimental geometries (Kayser *et al.*, 2014 ▸; Makita *et al.*, 2020 ▸) and slow processing speeds (Daurer *et al.*, 2020 ▸). In the absence of robust and computationally efficient wavefront sensing approaches that are compatible with the high duty cycle of next-generation facilities, the origin and characteristics of wavefront fluctuations at high repetition rates are poorly understood.

Addressing these limitations requires the development of fast, non-invasive and thermally robust diagnostic techniques capable of operating under the extreme conditions imposed by high-repetition-rate XFELs. In this work, we explore a passive approach to wavefront characterization based on X-ray speckle tracking (XST), in which macroscopic speckle patterns arising from beamline optics serve as intrinsic wavefront markers for phase retrieval (Mayo & Sexton, 2004 ▸; Morgan *et al.*, 2012 ▸; Bérujon *et al.*, 2012 ▸). Our XST-based method enables high-throughput sensor-free wavefront characterization at high repetition rates and represents a promising step toward real-time diagnostics for next-generation XFELs.

## 2. XST with a virtual reference

### 2.1. Principles of XST

Wavefields are characterized by their intensity and phase, which together define the local electromagnetic field strength and its evolution in space and time. While intensity can be measured directly, the phase must be inferred from measurements of intensity alone (Nugent, 2011 ▸; Paganin & Nugent, 2001 ▸). Solutions to this so-called *optical phase problem* are the central challenge of wavefront characterization. The wavefront is a conceptual surface of constant phase within the wavefield. The local direction of photon propagation is normal to this surface (Teague, 1983 ▸). Perturbations of the wavefront lead to local deflections in the direction of intensity propagation, which manifest as transverse displacements of intensity, relative to the unperturbed wavefront, when observed on a downstream detector.

XST exploits this effect to retrieve phase fluctuations from the distortion of a noise-like intensity background (Bérujon *et al.*, 2012 ▸). In the archetypal XST experiment, two intensity images are recorded: a reference intensity, *I*_0_, generated by the propagation of an X-ray wavefield through a speckle mask, and a subsequent intensity, *I*, recorded in the presence of an additional perturbing phase. The intensity at the detector, located in the optical near-field of the speckle mask, is a distorted projection of the speckle background and is given by a Fresnel convolution of the exit-surface wavefield (Paganin, 2013 ▸),
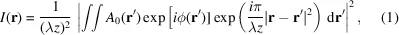
where 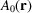 is the unperturbed complex amplitude at the exit surface of the speckle mask, 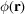 is the perturbing phase, λ is the X-ray wavelength, and *z* is the propagation distance from the speckle mask to the detector.

If the perturbing phase varies slowly in space it can be approximated locally as a first-order Taylor expansion,
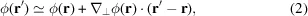
where ∇_⊥_ is the transverse gradient operator and is defined as 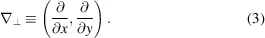
Substituting this expanded phase into the Fresnel integral in equation (1) yields the resulting intensity as a spatially shifted projection of the reference pattern, such that
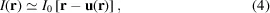
where 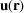 is the local displacement field that encodes the transverse phase gradient. In the near-field approximation, this displacement maps linearly to the gradient of the perturbing phase, such that
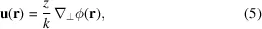
where *k* = 2π/λ is the X-ray wavevector.

Equation (5) provides a deterministic route to reconstructing phase shifts from local intensity displacements, forming the basis of gradient-based solutions to the optical phase problem. These methods infer the transverse flow of intensity along streamlines that are normal to the X-ray wavefront. In XST, the direction of propagation is extracted by tracking displacements in granular, noise-like speckle patterns that serve as spatially resolved wavefront markers. Such speckle is ubiquitous, and can arise when a partially coherent beam scatters from the quasi-random microstructure of common materials—such as paper, sandpaper, or ground glass—enabling the use of inexpensive speckle masks in place of dedicated optics (Zdora, 2018 ▸). When combined with the myriad robust and highly optimized computer vision algorithms for particle tracking (Banerjee *et al.*, 2025 ▸), XST provides a simple, non-interferometric method for wavefront characterization that is well suited to the emergent demands of high-repetition-rate XFELs.

### 2.2. Statistical wavefront analysis using a virtual reference

XST is typically employed to retrieve phase shifts introduced in an X-ray wavefield by an unknown object. These shifts are revealed as distortions of a static intensity background imprinted onto the X-ray wavefield by a dedicated speckle mask. The perturbing phase encodes local variations in the object’s refractive index which can be recovered by inversion of equation (5) (Paganin *et al.*, 2002 ▸). The robustness of these methods to experimental geometry, partial coherence and signal-to-noise ratio enables deterministic and computationally inexpensive phase-retrieval across a diverse range of applications in imaging (Morgan *et al.*, 2012 ▸; Zanette *et al.*, 2014 ▸; Paganin *et al.*, 2018 ▸; Alloo *et al.*, 2023 ▸) and metrology (Berujon *et al.*, 2013 ▸; Zdora *et al.*, 2017 ▸; Sawhney *et al.*, 2013 ▸).

The methodology outlined in Section 2.1 is agnostic to the specific origins of both the speckle background and the perturbing phase. It requires only that the location of origin of the X-ray speckle is known, and that it lies in the near-field of the X-ray detector. Here, we exploit this generality to adapt XST for the statistical characterization of wavefront fluctuations between XFEL pulses.

In this context, the wavefield product in equation (1) can be re-interpreted as the mean-field decomposition of the XFEL pulse ensemble. The mean-field component, *A*_0_, of this X-ray wavefield is time-independent, and can be defined as a static reference background, such that
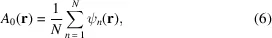
where ψ_*n*_(**r**) denotes the exit-surface wavefield of the *n*th pulse in an ensemble of *N* pulses. This formulation corresponds to an additive decomposition of the wavefield phase into a time-independent component contained within the mean field complex amplitude and a time-dependent phase disturbance. Such a description is consistent with existing models of shot-to-shot wavefront fluctuations observed at the European XFEL (Guest *et al.*, 2023 ▸).

**Figure 1:**
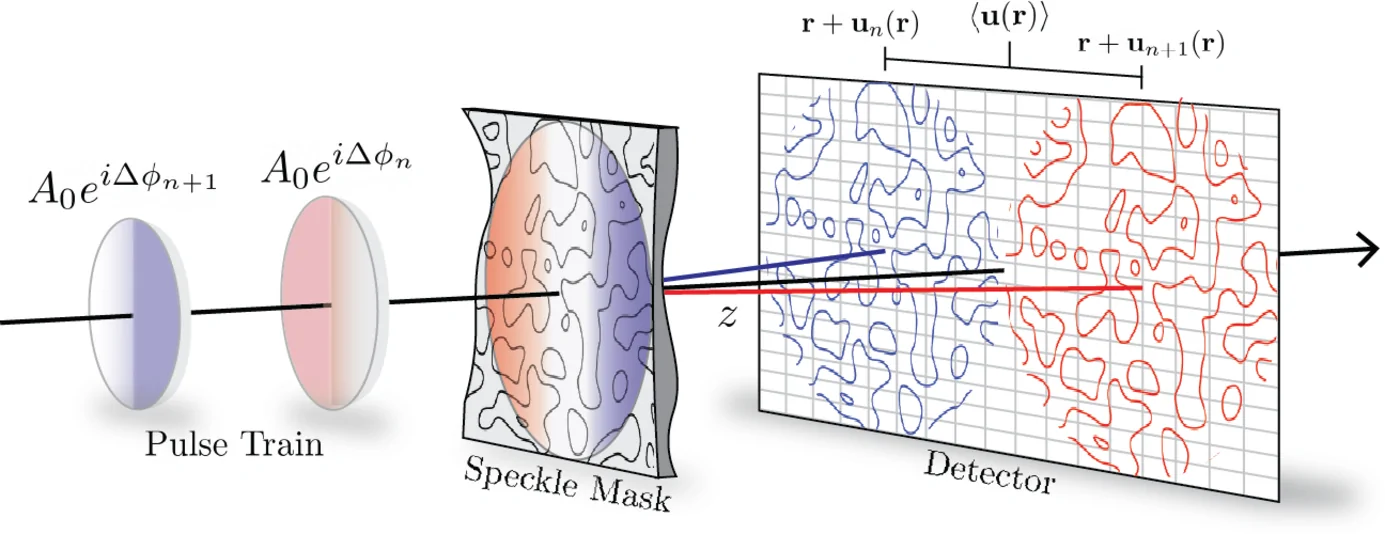
Conceptual illustration of the virtual-reference X-ray speckle tracking method. An X-ray pulse train with fluctuating phases illuminates a static speckle mask. A near-field detector records the corresponding set of transversally displaced speckle patterns. The magnitude of the displacement is proportional to the phase shift between pulses. We define a virtual reference at their midpoint, enabling a statistical reconstruction of wavefield fluctuations throughout the pulse train.

Under this interpretation, the observed speckle displacement arises from a *virtual* object that reflects the cumulative effect of transient angular instabilities in the source and beamline optics. These instabilities arise in addition to the mean-field component, which thus serves as a *virtual reference* against which fluctuations within and between individual pulse wavefronts can be compared. Our proposed approach to studying wavefront fluctuations is illustrated in Fig. 1 ▸.

Since the magnitude of fluctuations in X-ray phase has limited physical meaning in the present context, we thus consider this wavefront instability within the context of wavefront tilts, which manifest as changes in the *beam pointing* angle, 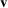 = 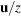. Let 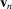 denote the spatial pointing variation of the *n*th pulse relative to an arbitrarily chosen reference wavefield. The ensemble-averaged beam pointing, which serves as our virtual reference, is thus defined as the mean angular deviation from this arbitrary reference frame, 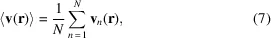
from which each pulse exhibits a relative pointing-angle error, given by 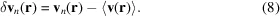
Assuming that variations in beam pointing are due to small angular deviations in the wavefront tilt, the local statistical variance quantifies the spread of these fluctuations, 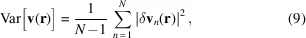
and can be expressed as the standard deviation of the wavefront pointing,
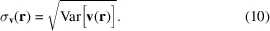
This statistical formulation quantifies the local wavefront stability of individual pulses relative to the ensemble-averaged wavefront, providing a general framework for characterizing shot-to-shot wavefront instabilities without requiring a dedicated reference intensity. We hereafter apply this framework to experimental data collected at the European XFEL, using the static speckle background generated by beamline optics to obtain robust high-throughput measurements of wavefront stability during operation at high repetition rates.

## 3. Method

**Figure 2:**
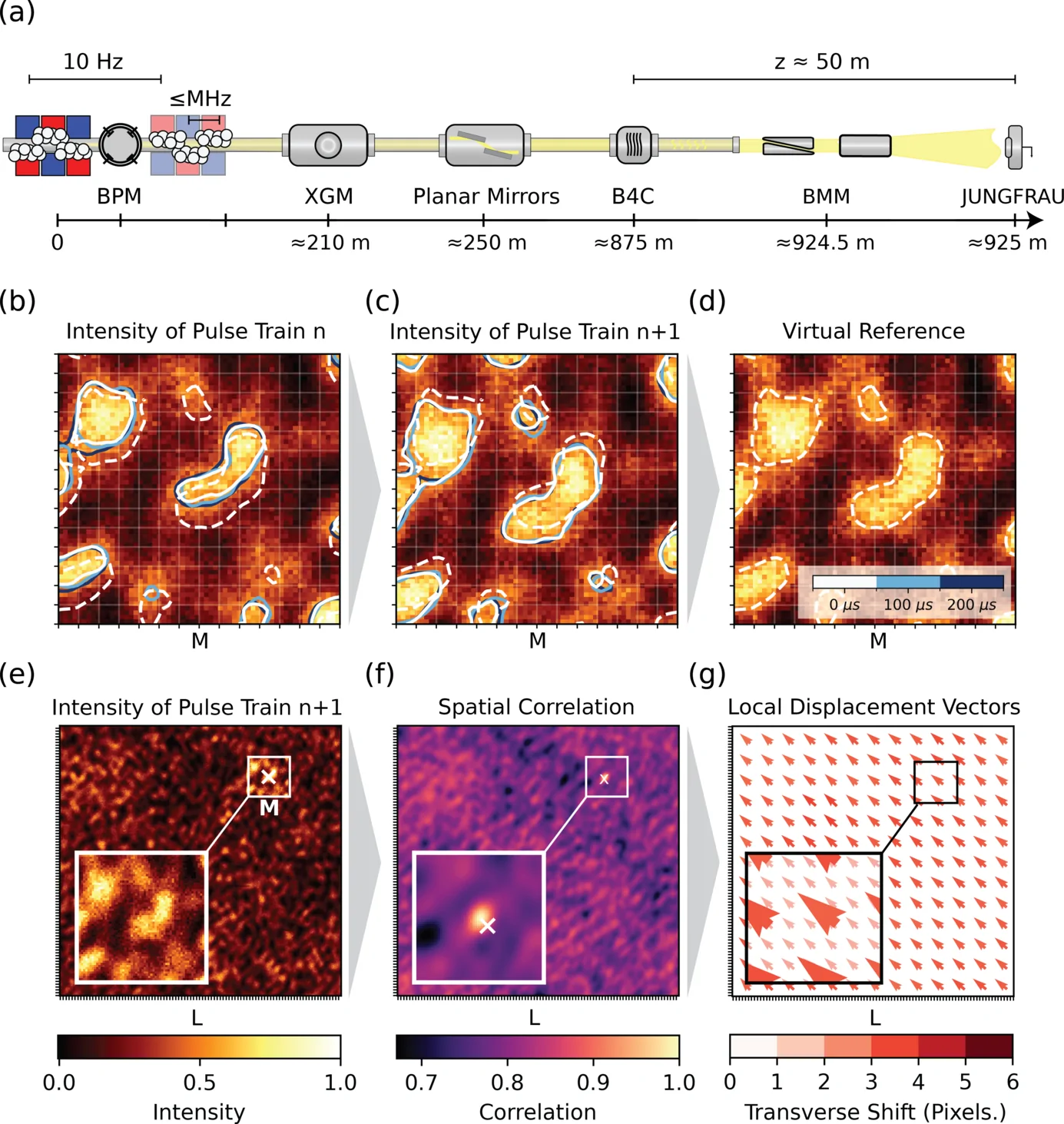
A summary of experiment and analysis methods. (*a*) Beamline and instrument geometry for XST measurements at the SPB/SFX instrument of the European XFEL. (*b*, *c*) Transverse shifts of X-ray speckles are monitored inter-train and intra-train (solid contours) and are used to construct a virtual reference describing the average speckle position. This virtual reference (dashed) is purely numerical, and is updated iteratively such that it lies at the midpoint of the speckle displacements in all preceding pulses. A representative reference intensity is presented in (*d*), which reflects the mean speckle location of speckle patterns (*b*, *c*). (*e*, *f*) The transverse shift of speckles from the perturbed location (crossmark) are determined from the spatial correlation between local subsets of the perturbed intensity and an arbitrary intensity reference. The distance between the perturbed location and correlation maxima are used to calculate (*g*) the local intensity displacement field, from which we extract beam pointing statistics.

XST measurements were performed at the the Single Particles, Clusters, and Biomolecules & Serial Femtosecond Crystallography (SPB/SFX) instrument of the European XFEL (Mancuso *et al.*, 2013 ▸). Collimated 10.65 keV pulses delivered to the instrument via a pair of horizontal offset mirrors (HOMs) were magnified using a Bragg microscope (BMM) (Vagovič *et al.*, 2013 ▸). In this magnified regime, the microscopic grain structure of beam conditioning optics introduce a static speckle background in the X-ray beam (Sinn *et al.*, 2019 ▸). Speckle patterns arising due to a 700 µm boron carbide attenuator were recorded on a JUNGFRAU-1M detector positioned *z* = 50 m downstream. Using the BMM, we achieved a ∼217× magnification of the X-ray beam, yielding an effective spatial resolution, *d***u**_min_ = 345 nm, and a corresponding sensitivity to pointing-angle fluctuations, *d***v**_min_ = *z*^−1^*d***u**_min_ = 6.9 nrad. Pulse delivery was synchronized to the 80.6 kHz detector frame rate (Sikorski *et al.*, 2023 ▸), with monitors of electron bunch position (BPM) and pulse energy (XGM) recorded simultaneously (Keil, 2016 ▸; Maltezopoulos *et al.*, 2019 ▸). A schematic of the experimental geometry is provided in Fig. 2 ▸(*a*).

Local wavefront fluctuations were quantified using a correlation-based speckle tracking approach (Morgan *et al.*, 2011 ▸; Morgan *et al.*, 2012 ▸). Transverse speckle displacements were determined from the maxima of the normalized cross-correlation between an arbitrarily selected reference frame of width *L* and perturbed intensity subset of width *M*
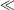
*L*. Wavefront fluctuations were resolved spatially by tracking subsets centred at *M*/2 intervals across the frame of interest, resulting in 13 × 13 intensity subsets per frame. A subset width of *M* = 60 pixels was selected to contain multiple speckle features in order to provide sufficient intensity variation to yield a unique and well defined correlation peak (Pan *et al.*, 2008 ▸). For a fixed reference frame, the computational cost of this approach scales with 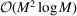 per intensity subset. Example intensity subsets and the subsequent analysis workflow are presented in Figs. 2 ▸(*b*)–2(*g*).

To isolate wavefront statistics representative of the inter-train, intra-train and single-shot timescales of the European XFEL, three independent virtual references were defined: (i) an inter-train reference (10 Hz) monitoring fluctuations defined relative to the full ensemble of recorded intensities, (ii) an intra-train reference (80.6 kHz) monitoring fluctuations defined relative to their corresponding train averages, and (iii) a single-shot reference monitoring fluctuations defined relative to the mean spatial error within each frame. The standard error of each reference is proportional to the inverse square of the number of recorded frames. After recording a total of 12448 pulses, we obtained virtual references with sub-nanoradian sensitivity on all three timescales. XST measurements relative to these references form the foundation for the statistical analysis presented in the following section.

## 4. Results

The wavefront statistics extracted here provide a quantitative basis for assessing wavefront stability at the entrance to the SPB/SFX instrument in relation to key operational parameters. In this section, we analyse the recorded speckle intensities to extract the magnitude, spatial structure and temporal dynamics of these fluctuations on the inter-train, intra-train and single-shot timescales. These statistics are interpreted in the context of known sources of beam instability, including mechanical vibrations of photon transport optics and shot-to-shot electron trajectory jitter.

### 4.1. Speckle tracking and wavefront statistics

The accuracy of speckle tracking algorithms critically depends on the characteristics of the recorded speckle patterns. The size of speckles, relative to the effective detector resolution, must be optimized to balance spatial wavefront sampling and local spatial uniqueness, while the speckle contrast, *C*, defined as the coefficient of variation of the X-ray intensity, describes the information content of the measurement. We begin by evaluating the quality and stability of speckle patterns recorded at the European XFEL to assess their suitability for wavefront characterization via XST.

**Figure 3:**
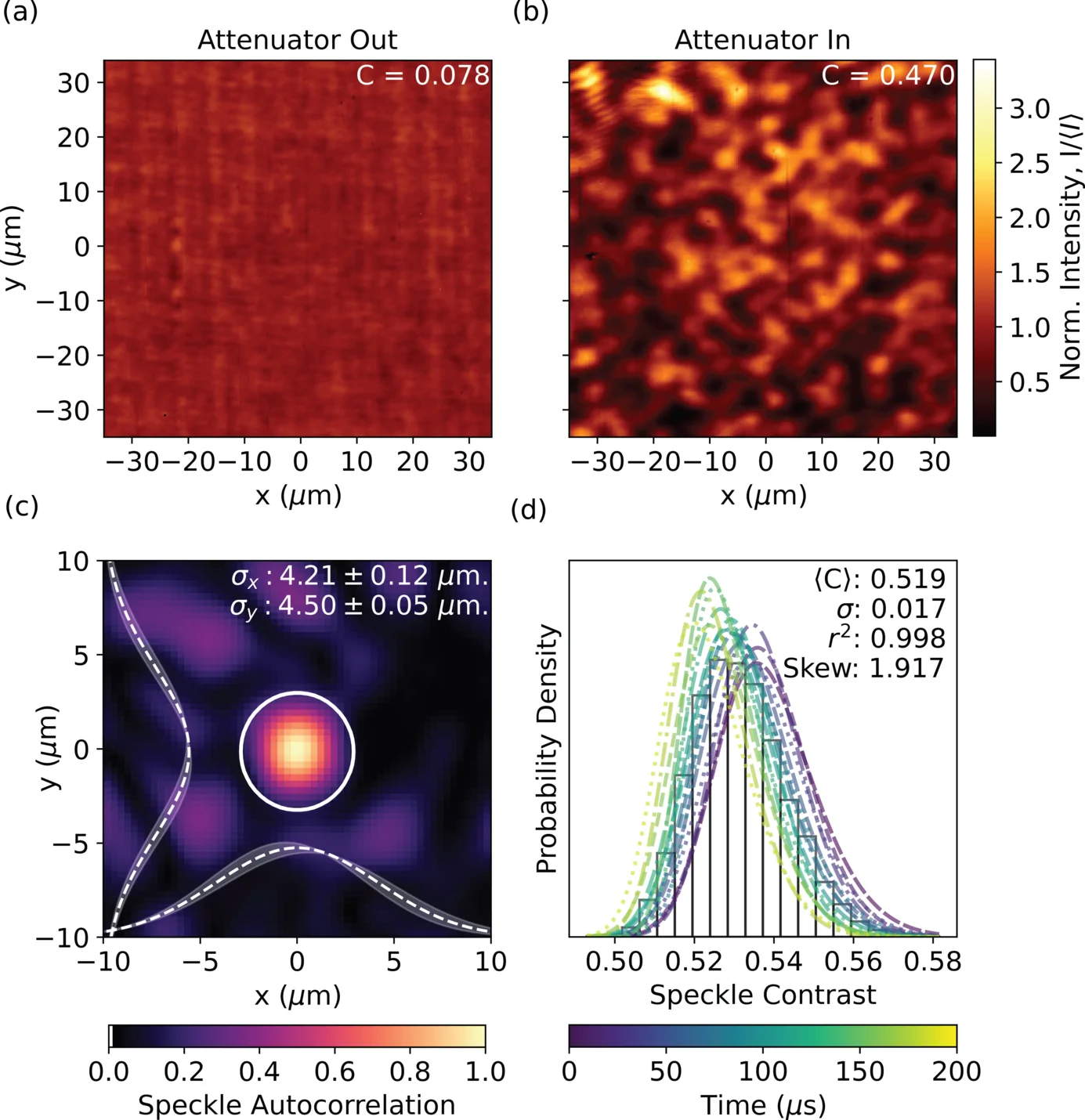
Evaluation of X-ray speckle intensity and characteristics. (*a*, *b*) Comparison of average pulse intensity before and after insertion of the solid attenuator. (*c*) The mean shift-corrected spatial autocorrelation of the speckle intensity. The solid contour maps 1σ width around the maxima of the ensemble average autocorrelation function. Transverse speckle widths, σ_*x*_ and σ_*y*_, were extracted from fits of the axial projections (dashed) of the ensemble. Shaded regions reflect the shot-to-shot deviation of the axial projections. (*d*) Pulse-indexed probability density of the speckle contrast, *C*, and skew normal fit of the ensemble probability density (histogram).

The characteristics of speckle recorded at the European XFEL are presented in Fig. 3 ▸, which demonstrates the emergence of a high contrast speckle background upon insertion of the solid attenuating foil. The transverse speckle widths, σ_*x*_ and σ_*y*_, obtained from Gaussian fits of the single-shot spatial intensity autocorrelation indicate a symmetric and isotropic speckle background, and the observed speckle width of ∼10 pixels is consistent with practical guidelines regarding speckle sampling (Zdora, 2018 ▸). The low shot-to-shot variance in speckle size indicates a residual wavefront curvature multiple orders of magnitude smaller than the wavefront phase (see Appendix *A*[App appa]) and thus supports the validity of the smooth-phase approximation employed in equation (2).

The thermal stability of the speckle-generating solid attenuator is critical to the accurate interpretation of wavefront stability. While the near-field speckle tracking approach relaxes thermal stability requirements in comparison with deterministic phase encoders such as gratings or crystals, it nonetheless remains important to ensure that variations in the observed speckle intensities do not arise from thermoelastic deformation of the speckle-generating material. This is reflected here by the predominantly stable speckle size and morphology extracted from the speckle autocorrelation function and its axial projections, which indicate that the high melting point, low thermal expansion coefficient, and comparatively high thermal diffusivity of boron carbide are sufficient to suppress significant thermal strain and lensing under the present heat loads. Combined with the narrow shot-to-shot contrast distribution illustrated in Fig. 3 ▸(*d*), these measurements indicate stable, high-quality speckle that is suitable for high-fidelity wavefront metrology (Zdora, 2018 ▸).

**Figure 4:**
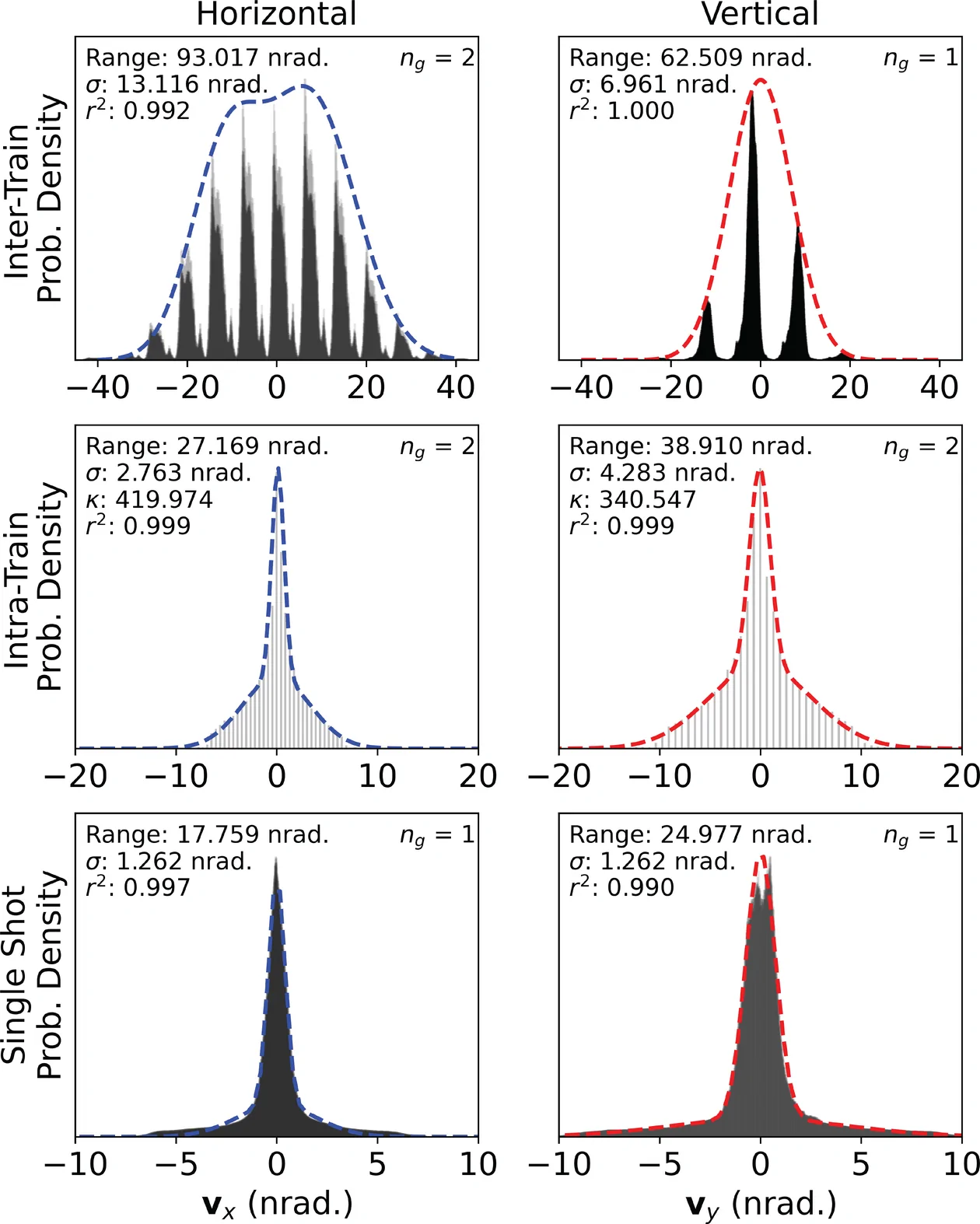
Probability density distributions of transverse pointing-angle fluctuations at the entrance to the SPB/SFX instrument of the European XFEL. Histograms show the distribution of pointing deviations after centering on the inter-train, intra-train and single-shot mean angles (virtual references), where κ denotes the kurtosis of the intra-train pointing-angle distributions. Shot-to-shot pointing-angle measurements reflect the spatial averaging of discrete speckle displacements derived from finite detector sampling. As the intrinsic single-shot pointing uncertainty is substantially smaller than the effective discretization interval of the estimator, the inter-train distributions exhibit an apparent multi-peak structure. Gaussian mixture models of order *n*_g_ are therefore used to capture the underlying envelope of these distributions and estimate their overall range and variance.

A qualitative evaluation of the speckle intensities shown in Figs. 2 ▸(*b*)–2(*c*) provides a preliminary insight into the nature of the underlying wavefront displacements. The observed displacements manifest as rigid transverse shifts of the entire speckle pattern without apparent deformation, suggesting that the associated wavefront errors vary slowly in space. This visual assessment also offers an early indication of the relative magnitude of pointing instabilities within and between pulse trains, *i.e.* that individual speckles exhibit larger displacements between trains than within them. To quantify these observations, we compare the first-order statistics of wavefront fluctuations arising on the inter-train, intra-train and single-shot timescales in Fig. 4 ▸.

This statistical analysis reveals a temporal hierarchy of wavefront fluctuations at the entrance to the SPB/SFX instrument. Wavefront fluctuations grow with increasing sampling interval, reflecting the accumulation of slow drifts and instabilities over time. The beam is most stable on a single-shot basis, with displacements becoming progressively larger on intra-train and inter-train timescales. These observations are consistent with previous reports of beam position jitter and coherence at the European XFEL (Guest *et al.*, 2022 ▸; Dallari *et al.*, 2022 ▸).

The corresponding pointing-angle distributions illustrate distinct statistical properties on each of the timescales evaluated. On the single-shot timescale, the probability density of the transverse beam pointing-angle distributions are described by narrow normal distributions, indicating that spatial fluctuations within individual pulse wavefronts are small isotropic deviations of an otherwise planar wavefront tilt. The magnitude and direction of these tilts vary from shot-to-shot, leading to increased pointing-angle variance on the inter-train and intra-train timescales, and allude to extrinsic sources of instability evolving on timescales far longer than the duration of a single pulse. The inter-train statistics, which represent the full ensemble of wavefront fluctuations, exhibit a broad, bimodal horizontal distribution, indicative of slow horizontal drifts or oscillations, which are absent in the vertical direction. This asymmetry is reversed intra-train, where the high kurtosis of the distribution (κ ≫ 3) is indicative of leptokurtic beam pointing statistics describing transient angular deviations around an otherwise stable beam path.

### 4.2. Origins of wavefront fluctuation

The marked variation in the shape and width of the distribution of wavefront fluctuations on the inter- and intra-train timescales alludes to the existence of multiple, independent sources of wavefront instability with distinct temporal signatures. We subsequently explore this hypothesis by evaluating the temporal characteristics of these fluctuating wavefront statistics with respect to known sources of instability in the accelerator and beamline optics.

**Figure 5:**
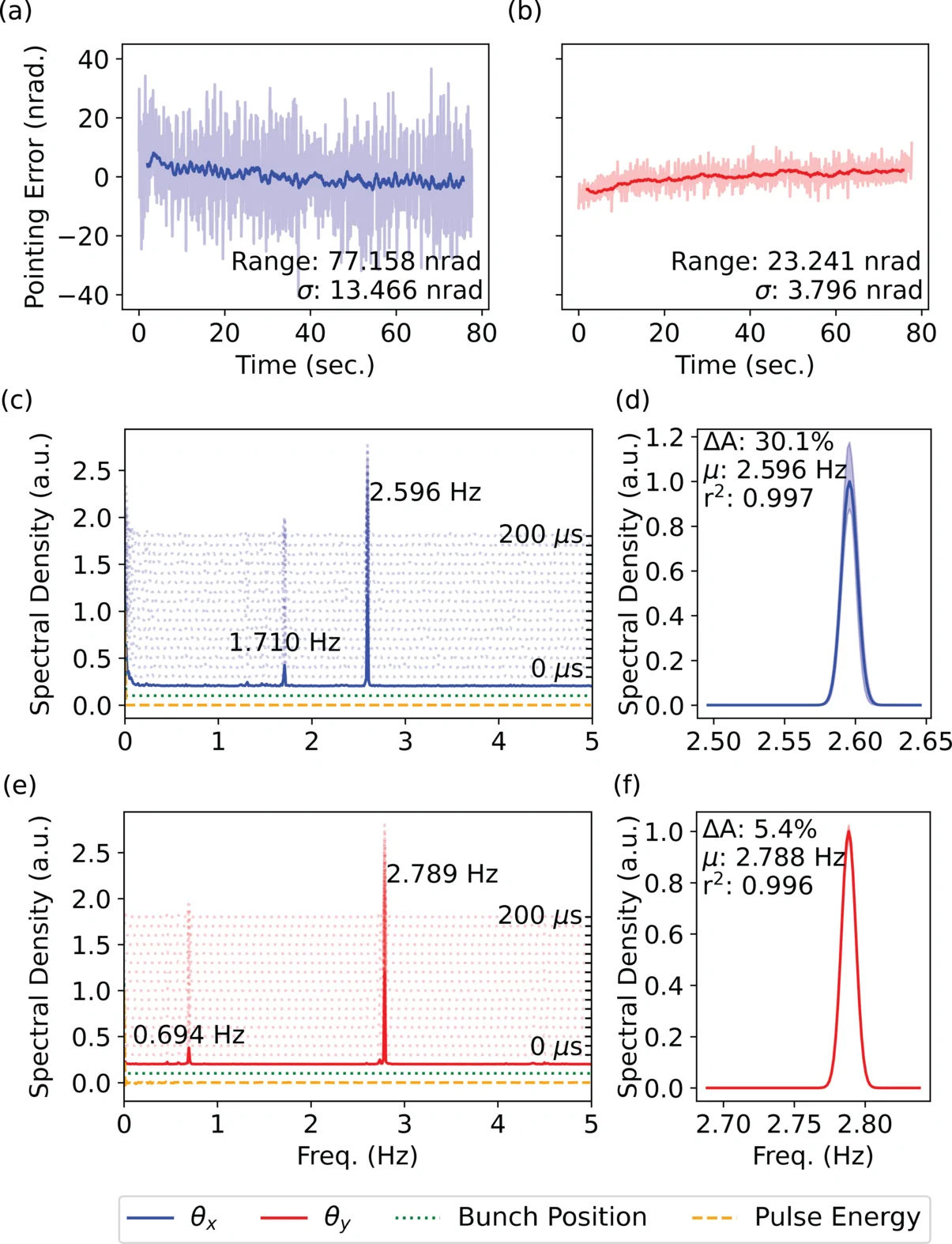
Fourier analysis of inter-train pointing-angle fluctuations at the European XFEL. (*a*, *b*) Temporal evolution of horizontal and vertical inter-train pointing errors, with shaded regions indicating the range of low-frequency oscillations, and their corresponding (*c*, *e*) frequency-domain representations. The mean (solid) and pulse-resolved (dotted) spectra are presented alongside frequency domain representations of simultaneous fluctuations in electron bunch position and pulse energy, as measured on the BPM and XGM, respectively. Magnified fits of the dominant Fourier modes are presented in panels (*d*, *f*), with the shaded trace quantifying the amplitude variation across the pulse train.

The inter-train statistics in Fig. 4 ▸ exhibit broad, asymmetric and multi-modal behaviour, with wavefront fluctuations most pronounced in the horizontal direction. This asymmetry is consistent with the large horizontal aperture of the photon transport mirrors, which engender an increased susceptibility to structural resonances, providing a strong indication that the observed inter-train fluctuations are of mechanical origin. Mechanical vibrations are a persistent challenge in optomechanical systems, and a likely contributor to wavefront instability at the European XFEL due to the long optical path between photon transport optics and the scientific instruments. To evaluate the role of these vibrational instabilities on the stability of the X-ray wavefront, we analyse the temporal structure of the train-averaged pointing-angles in Fig. 5 ▸.

This frequency-domain representation isolates periodic wavefront fluctuations, revealing low-frequency instabilities driving wavefront fluctuations on the inter-train timescale. Our analysis reveals that the dominant source of these fluctuations emerge as 2.5–2.8 Hz periodic oscillations in beam pointing-angle. These frequencies coincide with known oscillatory modes of the European XFEL photon transport optics which have been observed via photon diagnostic measurements (Freund, 2024 ▸) as well as independent vibration sensors (Sinn *et al.*, 2022 ▸; Schmidtchen *et al.*, 2023 ▸). The observed spectral features are narrowband, stationary in frequency, and remain coherent across the intra-train dimension, consistent with stable mechanical vibrations rather than stochastic or thermally driven effects. While additional spectral peaks are observed near the 1/2 and 1/4 harmonics of the primary components, no corresponding periodicity is observed in the electron bunch position or X-ray pulse energy, both of which are measured upstream of the photon transport optics.

The variation in the amplitude of the dominant Fourier modes across the pulse index—particularly in the horizontal direction—reveals the existence of fast intra-train dynamics which modulate the effective strength of the underlying mechanical oscillations. In addition to the skewed intra-train speckle contrast distribution [Fig. 3 ▸(*d*)], and kurtosis observed in the intra-train pointing-angle distributions (Fig. 4 ▸), this time-evolving wavefront variance indicates that fluctuations observed within each pulse train arise from structured, temporally correlated components.

**Figure 6:**
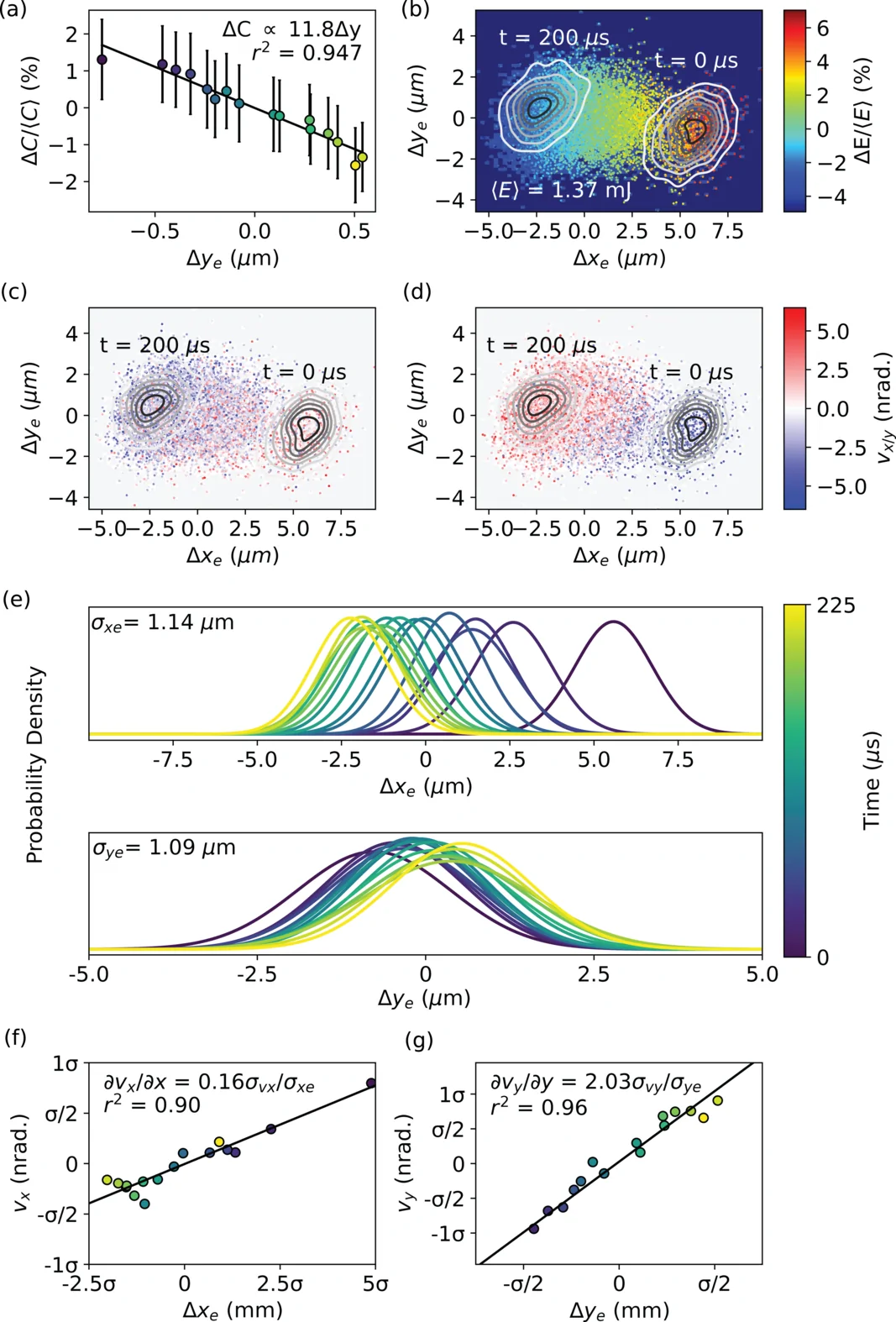
Influence of electron bunch trajectory on X-ray wavefront. (*a*) Inter-train reductions in speckle-contrast are linearly correlated with vertical electron bunch displacement. (*b*) The spatial distribution of corresponding pulse energies, where contours illustrate the probability density of electron bunch positions, *x*_e_ and *y*_e_, at the undulator source plane at two times throughout the pulse train (0 µs and 200 µs). The joint distribution of electron bunch positions and (*c*) horizontal and (*d*) vertical transverse tilts reveal an intra-train evolution of bunch position that is correlated with beam pointing. (*e*) This evolution is presented in (*e*), which illustrates the projected distributions of fluctuations in electron bunch position in the transverse plane. (*f*, *g*) These fluctuations are linearly correlated with wavefront tilt in the horizontal and vertical directions, respectively.

Changes in electron bunch trajectory and source position are known contributors to FEL instability at high repetition rates (Hellert *et al.*, 2017*b* ▸) and provide one potential explanation for the observed intra-train dynamics. Such instabilities are known to degrade lasing efficiency, increase gain length and lead to fluctuations in X-ray beam pointing (Tanaka *et al.*, 2004 ▸). To evaluate the impact of this orbit instability on the X-ray wavefronts observed at scientific instruments of the European XFEL, we hereafter analyse intra-train variations in pulse energy and wavefront pointing with respect to the corresponding electron bunch position at the nominal FEL source location. These results are presented in Fig. 6 ▸, where we evaluate the intra-train evolution of wavefront tilt and its relationship to electron bunch motion and pulse energy.

We observe a clear, pulse-resolved correlation between electron beam motion and both pulse energy and wavefront tilt. This observation indicates that intra-train wavefront fluctuations are tightly coupled to variations in electron trajectory on a shot-to-shot basis. Transverse drifts of the electron bunch at the nominal FEL source location evolve over the 225 µs pulse train and lead to a reduction in measured pulse energy, consistent with the intra-train degradation of speckle contrast shown in Fig. 3 ▸(*d*). These drifts—manifesting as systematic displacements of an otherwise symmetric electron distribution—are linearly correlated with fluctuations in the transverse pointing-angle. The structure of this correlation is highly reproducible across pulse trains, repeating with the 10 Hz machine cycle, as shown by the narrow, train-synchronous distribution of pulse-indexed electron positions. These results, in conjunction with the periodic wavefront instabilities observed on the inter-train timescale, establish a causal relationship between operational parameters and wavefront statistics at the European XFEL.

## 5. Discussion and outlook

In this study, we developed and implemented a reference- and sensor-free approach to wavefront characterization at the SPB/SFX instrument of the European XFEL. By circumventing the limitations of traditional diagnostics, our method enables shot-to-shot characterization of XFEL pulses at high repetition rates. This was achieved through a statistical formulation of XST, in which virtual reference wavefields were constructed at three operating frequencies, allowing us to resolve the hierarchical temporal structure of wavefront fluctuations at the European XFEL without the need for a dedicated reference. This demonstration represents a significant step toward routine wavefront diagnostics at next-generation XFEL facilities.

Our results reveal that shot-to-shot wavefront fluctuations at the entrance to the SPB/SFX instrument of the European XFEL are dominated by planar wavefront tilts. The magnitude and variance of these tilts were found to grow over time. A statistical analysis of the corresponding pointing-angles suggests two principal mechanisms degrading the shot-to-shot stability of the X-ray beam: high-frequency jitter correlated with intra-train electron trajectory fluctuations, and low-frequency drifts which coincide with known mechanical oscillation modes of photon transport optics. Collectively, these mechanisms describe a large proportion of the observed wavefront fluctuations. Our findings provide new insight into the origins and characteristics of wavefront fluctuations during high-repetition-rate operation at the European XFEL and can be used to construct heuristic guidelines for experiment design and data analysis.

### 5.1. Methodological extensions, limitations and opportunities

The simplicity and computational efficiency of our approach are well suited for routine diagnostic measurements of X-ray wavefront stability during photon transport at next-generation XFELs. This capability stems from two key conceptual advances. Firstly, by re-conceptualizing intrinsic beamline speckle as a static wavefront marker, we eliminate the need for dedicated wavefront sensing optics. Secondly, by introducing the notion of a virtual reference wavefield, we establish a generalisable framework for spatiotemporal statistical analysis of wavefront fluctuations across a range of operational timescales. In combination, these developments enable fast, deterministic and non-invasive phase retrieval via established XST techniques.

The capacity to measure spatially resolved wavefront statistics at high repetition rates with minimal operational and computational overhead enables new opportunities for experiment control. Existing online analysis infrastructure at the European XFEL supports real-time processing and display of megapixel-scale detector data acquired at repetition rates up to 4.5 MHz (Schmidt *et al.*, 2024 ▸). Comparably process-intensive correlation analysis pipelines have already been implemented online (Jakobsen *et al.*, 2025 ▸), including single-shot correlation analysis between pulse trains. These established analysis frameworks could therefore support real-time evaluation and live display of wavefront stability as a photon diagnostic. Such capabilities provide a route toward experiment-driven diagnosis of single-shot wavefront errors and their underlying sources, where online monitoring of wavefront statistics could enable wavefront tuning and optimization during both facility operation and experiment.

Nonetheless, the accuracy and performance of our approach can be further improved by leveraging the extensive optimization and development of XST for applications at synchrotron radiation facilities. Methodological and theoretical adaptations of the correlation-based approach utilized here have demonstrated improved angular sensitivity and noise robustness (Zdora *et al.*, 2018 ▸; Qiao *et al.*, 2020 ▸), and could be employed to achieve sub-pixel spatial resolutions at the expense of computational efficiency. Alternatively, substitution of the correlation-based XST algorithm with implicit speckle-tracking (Paganin *et al.*, 2018 ▸) or machine-learning approaches (Wang *et al.*, 2023 ▸) could significantly reduce computational load and subsequently increase the rate of wavefront characterization. A comprehensive review of these methods and a contextualization of their relative merits has been given by Celestre *et al.* (2025 ▸).

The general implementation of our method as a routine wavefront diagnostic is subject to geometric constraints. While we here assume smooth shot-to-shot wavefront variations, large fluctuations in the effective longitudinal FEL source position can lead to non-negligible changes in the shot-to-shot wavefront curvature which may invalidate this assumption (Rutishauser *et al.*, 2012 ▸). Under such circumstances, equation (2) can be extended to additionally retrieve fluctuations in the phase curvature term, 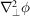, as has been demonstrated in XST applications in imaging (Berujon *et al.*, 2015 ▸). A related challenge can arise in focused beamline geometries, where the shortened optical near-field—which scales with the inverse of the square of the magnification—can limit angular resolution. In these regimes, iterative phase retrieval methods may be preferable at the expense of computational efficiency (Morgan *et al.*, 2020 ▸), though the statistical framework introduced here remains valid. Absolute wavefield recovery under such conditions is also permitted by prior characterization of the speckle-generating attenuator (Berujon *et al.*, 2015 ▸; Zdora *et al.*, 2018 ▸; Lee *et al.*, 2023 ▸).

Finally, our results demonstrate that the high-contrast X-ray speckle generated by beamline conditioning optics can be exploited in XST applications beyond conventional wavefront sensing. Our reference-free approach is particularly relevant for at-wavelength metrology, where dynamic aberrations induced by high heat loads remain challenging to characterize *in situ* (Petrov *et al.*, 2022 ▸). These aberrations pose a critical limitation for dispersive optical elements in split-and-delay lines (Rysov *et al.*, 2019 ▸), where our wavefront diagnostics can enable the thermal strain of beam-splitting optics to be monitored during high-repetition-rate operation. In imaging, the use of beamline speckle could enhance sensitivity to phase contrast, enabling quantitative access to sample composition and dynamics in pump–probe and time-resolved experiments. This opportunity is particularly relevant to the study of dense and heterogeneous materials, such as inertial confinement fusion targets, where extracting quantitative information from propagated phase contrast alone remains challenging (Montgomery, 2023 ▸). With the planned development of XFEL beamlines operating at ever-increasing photon energies—accompanied by extended near-field distances and reduced scattering cross-sections—the relevance and applicability of our approach is expected to grow.

### 5.2. Interpretation of wavefront instabilities

This study identifies the principal sources of wavefront instability retrieved using our approach and evaluates their implications for experiment operation and data analysis. Our results implicate mechanical oscillations of photon transport optics and electron bunch trajectory instabilities as the dominant contributors on the inter- and intra-train timescales, respectively. These findings highlight opportunities for improving wavefront stability through both physical and analytical interventions, and emphasize the need to address optical and accelerator-based sources of instability to preserve high shot-to-shot beam quality in high-repetition-rate XFEL experiments.

The instabilities observed here reflect long-standing challenges at XFEL and synchrotron light sources and persist despite mitigation efforts such as passive vibration damping (Altarelli, 2006 ▸) and electron-bunch orbit feedback (Keil, 2016 ▸). At the European XFEL, their impact is amplified due to long distances between the FEL source, photon transport optics and the scientific instruments, which results in nano­radian sensitivity to fluctuations in beam pointing during experiments. The predominant sources of these instabilities are known to arise from a broad range of largely unavoidable environmental factors—including local traffic, seismic motion, tidal forces, and vibrations from electrical, cooling or vacuum infrastructures (Li *et al.*, 2011 ▸). For as long as active optical corrections are challenging to apply to correct the vibrational motion on the observed time and length scales (Koehlenbeck *et al.*, 2025 ▸), the complete suppression of wavefront fluctuations in experiment remains unlikely.

As a consequence, it is critical that insights from studies such as the present guide future experimental and data analysis strategies. By providing direct access to wavefront statistics, our virtual-reference approach to XST establishes a physical basis for data classification and filtering. Our measurements reveal cyclostationary wavefront statistics arising from changes in beam pointing-angle due to the oscillation of photon-transport optics, such that processing steps that assume stationarity—including normalization, background estimation and merging—are only valid modulo the oscillation period (Gardner *et al.*, 2006 ▸). Sorting data into narrow phase bands of the dominant oscillation frequency can therefore yield a series of mutually incoherent subsets, each with lower internal variance than the full ensemble. Such wavefront-aware diagnostic strategies are essential for maximizing the potential of techniques that aggregate large numbers of weak and heterogeneous measurements over long durations, such as Single Particle Imaging and Serial Femtosecond Crystallography, which utilize the high repetition rate of the European XFEL to sample a sparse and randomly temporally distributed orientation space (Ayyer *et al.*, 2019 ▸; Darmanin *et al.*, 2016 ▸; Holmes *et al.*, 2022 ▸; Poudyal *et al.*, 2020 ▸).

The capacity of our method to resolve distinct fluctuation modes is nonetheless limited by the temporal bandwidth of the detector system and the finite repetition rate of the X-ray beam. The 80.6 kHz frame rate of the JUNGFRAU detector corresponds to a Nyquist frequency of 40.3 kHz, such that MHz rate dynamics can only be measured as ensemble variance between sampled pulses. More fundamentally, the burst-mode operation of the European XFEL imposes an intrinsic hierarchy of discrete sampling scales, with inter-train dynamics observed at 10 Hz and intra-train dynamics sampled at up to 4.5 MHz. While the underlying accelerator and photon transport system supports a continuum of potential fluctuations, ranging from long-timescale environmental variations occurring over days (Czwalinna *et al.*, 2021 ▸) to the GHz frequencies of the machine clock and RF systems, each X-ray pulse represents only a discrete realization of the machine state.

It follows that independent diagnostics monitoring the vibrational spectra of photon-transport optics and drifts of the electron-beam orbit are essential to identifying latent correlations between facility operation and wavefront stability. By constructing surrogate estimators of the pulse wavefront (Grech *et al.*, 2023 ▸; Ferreira de Lima *et al.*, 2024 ▸; Bishara *et al.*, 2025 ▸), such complementary proxy measurements could be used to extract signal from noise in the persistent presence of wavefront instability at next-generation facilities.

## 6. Conclusion

In this work, we have introduced and experimentally demonstrated a method for shot-to-shot wavefront characterization at a high-repetition-rate XFEL. By developing a virtual-reference approach to X-ray speckle tracking, we circumvent the need for dedicated wavefront optics and provide a robust, non-interferometric wavefront sensing method compatible with the high repetition rate of next-generation facilities. Our implementation enables the extraction of spatially resolved wavefront fluctuations, where we forego the requirement of a dedicated wavefront sensor by utilizing the intrinsic speckle generated by beamline optics. Applying this method at the European XFEL, we have identified a temporal hierarchy of wavefront fluctuations with distinct statistical properties, providing insight on the underlying electronic and mechanical sources of instability. Our results constitute the first demonstration of high-repetition-rate wavefront characterization at a next-generation facility, and provide a framework for mitigating these fluctuations in experiment and data analysis.

## Data Availability

The speckle tracking source code will be uploaded to a public repository with demonstration upon publication of the manuscript. Raw data recorded at the European XFEL (DOI: https://doi.org/10.22003/XFEL.EU-DATA-005056-00) will be made publicly available following the three-year embargo period ending 27-05-2026.

## Appendix A Validity of the smooth-phase approximation

The X-ray phase can be written as a Taylor expansion of the general form

If the X-ray phase is a smoothly varying function in space, this general form can be truncated to first order, yielding the smooth-phase approximation presented in equation (2). This reduced representation of the X-ray phase is underpinned by the assumption that higher-order terms in the phase expansion are negligible compared with the local wavefront tilt. Physically, this corresponds to the requirement that the wavefront curvature is weak over the spatial scale of interest. For a paraxial spherical wavefront with radius of curvature *R*,

such that the first- and second-order derivatives in the Taylor expansion of the phase scale with the wavefront curvature, 1/*R*,

The smooth-phase approximation therefore requires that curvature-induced phase variations remain small compared with the dominant linear phase gradient over the characteristic speckle width, such that

Our virtual reference approach is sensitive to the phase difference between pulses, and thus residual curvature fluctuations of the X-ray wavefront. This curvature manifests as the shot-to-shot contraction or dilation of X-ray speckle, such that the variance of the speckle width extracted from the autocorrelation of the speckle intensity [Fig. 3 ▸(*c*) of the main text] can be used to estimate the magnitude of residual wavefront curvature fluctuations.

Assuming an approximately Gaussian FEL source of wavelength λ and waist width σ_0_, the wavefront curvature is given analytically as (Saleh & Teich, 2019 ▸)

where *z*_R_ is the Rayleigh length, such that

For an FEL source with an effective beam waist σ_0_ = 39.26 µm at 10.65 keV (Guest *et al.*, 2023 ▸), substituting equation (17) into equation (16) yields an absolute wavefront curvature estimate of 0.0011 m^−1^ in the plane of the B_4_C attenuator.

We observe relative fluctuations in speckle size that are most pronounced in the horizontal direction, σ_*x*_ = 4.21 ± 0.12 µm, yielding a shot-to-shot distribution of speckle sizes with a standard deviation of 2.77%. This corresponds to a standard residual curvature of 3.232 × 10^−5^ m^−1^ and gives an estimate of the residual quadratic phase contribution as

Equivalently, the residual curvature produces an angular variation across an individual speckle of

This curvature-induced phase variation is substantially smaller than unity and remains small compared with the phase gradients associated with the nanoradian-scale wavefront tilts analysed in Section 4. The observed speckle-size fluctuations therefore indicate a measurable residual curvature component, but not a breakdown of the first-order smooth-phase approximation over the local speckle length scale.
